# Nicked-site substrates for a serine recombinase reveal enzyme–DNA communications and an essential tethering role of covalent enzyme–DNA linkages

**DOI:** 10.1093/nar/gkv521

**Published:** 2015-05-18

**Authors:** Femi J. Olorunniji, Arlene L. McPherson, Hania J. Pavlou, Michael J. McIlwraith, John A. Brazier, Richard Cosstick, W. Marshall Stark

**Affiliations:** 1 Institute of Molecular, Cell and Systems Biology, University of Glasgow, Bower Building, Glasgow G12 8QQ, UK; 2 Department of Physiology, Anatomy and Genetics, University of Oxford, Sherrington Building, Parks Road, Oxford OX1 3PT, UK; 3 Beatson Institute for Cancer Research, Switchback Road, Bearsden, Glasgow G61 1BD, UK; 4 Reading School of Pharmacy, University of Reading, Whiteknights, Reading, Berkshire, RG6 6AD, UK; 5 Department of Chemistry, University of Liverpool, Crown Street, Liverpool, L69 7ZD, UK

## Abstract

To analyse the mechanism and kinetics of DNA strand cleavages catalysed by the serine recombinase Tn*3* resolvase, we made modified recombination sites with a single-strand nick in one of the two DNA strands. Resolvase acting on these sites cleaves the intact strand very rapidly, giving an abnormal half-site product which accumulates. We propose that these reactions mimic second-strand cleavage of an unmodified site. Cleavage occurs in a synapse of two sites, held together by a resolvase tetramer; cleavage at one site stimulates cleavage at the partner site. After cleavage of a nicked-site substrate, the half-site that is not covalently linked to a resolvase subunit dissociates rapidly from the synapse, destabilizing the entire complex. The covalent resolvase–DNA linkages in the natural reaction intermediate thus perform an essential DNA-tethering function. Chemical modifications of a nicked-site substrate at the positions of the scissile phosphodiesters result in abolition or inhibition of resolvase-mediated cleavage and effects on resolvase binding and synapsis, providing insight into the serine recombinase catalytic mechanism and how resolvase interacts with the substrate DNA.

## INTRODUCTION

**Figure 1.**
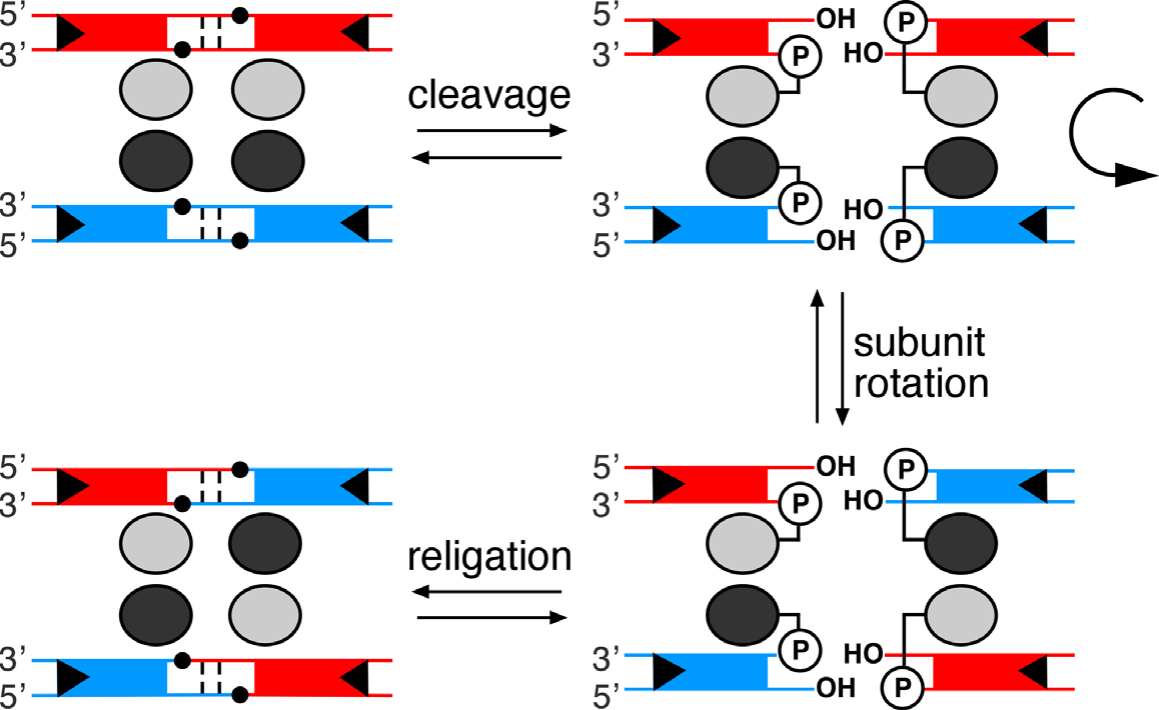
Strand exchange mechanism for serine recombinases. The reaction occurs in a synaptic complex comprising four resolvase subunits (ovals) and two crossover sites (site I) (tramlines). The small black circles mark the positions of the phosphodiester groups attacked by the hydroxyl group of the nucleophilic S10 residue (shown as circled P in the cleaved intermediates).

In site-specific recombination, the DNA double helix is cleaved by breaking both strands at two precisely defined positions, and the broken ends are rejoined to new partners. The process is catalysed by a recombinase enzyme. Two large groups of recombinases known as the tyrosine recombinases and the serine recombinases have been the subject of extensive biochemical and structural analysis (1). Serine recombinases (2) exchange DNA strands between two ‘crossover sites’ (typically ∼30 bp long). Each crossover site binds a recombinase dimer, and two crossover sites are brought together by dimer–dimer interactions to form a synaptic tetramer. Double-strand cleavage at specific phosphodiester bonds close to the centre of each site results in an intermediate with recombinase subunits attached to each 5′ DNA end via a phosphodiester with the active site serine. Following a rotation-like exchange of the positions of the cleaved half-sites, the strands are rejoined to form recombinant sites (1,3) (Figure 1).

There are thus eight chemical steps in a complete site-specific recombination reaction: four strand cleavages and four ligations. For efficient, precise product formation, these steps must be coordinated. In the case of serine recombinase-mediated recombination, all four strands must be cleaved before strand exchange can take place, and all four strand breaks must then be efficiently re-sealed to form undamaged recombinant sites. The apparent simplicity of the overall recombination reaction thus disguises a complex, choreographed series of events, whose kinetics are still largely unknown. Some questions we would like to answer are: Does strand cleavage require formation of a synaptic tetramer? What is the rate of each of the strand cleavage and ligation steps? To what extent are the individual steps reversible? How are the individual steps coordinated? One strategy to advance our understanding aims to dissect the reaction by using DNA substrates with chemical modifications that interfere with specific steps. These modified substrates can also provide information on the specific interactions of the recombinase with its target sites, which could not be gained from reactions with ordinary substrates. Here we analyse the mechanism of the serine recombinase Tn*3* resolvase (4), using modified substrates containing a break (‘nick’) in one strand of the recombination site.

## MATERIALS AND METHODS

### DNA oligonucleotides and resolvase

Standard, phosphorothioate-modified and 5′-phosphorylated DNA oligonucleotides were obtained from Eurofins-MWG. Oligonucleotides with methylphosphonate modifications were from Eurogentec Inc. Oligonucleotides containing 3′-phosphorothiolate modifications were synthesized as previously described (5,6). All oligonucleotides were purified by HPLC and/or PAGE. Gels and buffers for purification of phosphorothiolate-containing oligonucleotides contained 0.5-mM dithiothreitol (DTT).

Oligonucleotides with a 2′, 3′-dideoxynucleotide at the 3′ terminus were prepared by addition of the dideoxynucleotide with terminal transferase (New England Biolabs). The reaction was at 37°C for 30 min in a buffer containing 50 mM potassium acetate, 20 mM Tris-acetate, 10 mM magnesium acetate, 0.25 mM cobalt chloride, 0.25 mM ddTTP (Amersham Biosciences) and 1.2 μM purified oligonucleotide. The reaction products were then purified by PAGE.

The resolvase proteins used in this work were (i) wild-type (WT) Tn*3* resolvase; (ii) NM resolvase, an ‘activated’ variant with six mutations from WT Tn*3* resolvase (R2A, E56K, G101S, D102Y, M103I and Q105L); (iii) NM S10A, a catalytically inactive variant, with all the mutations in NM resolvase and the additional mutation S10A. Overexpression and purification of these proteins were as described in (7).

### *In vitro* assays

**Figure 2.**
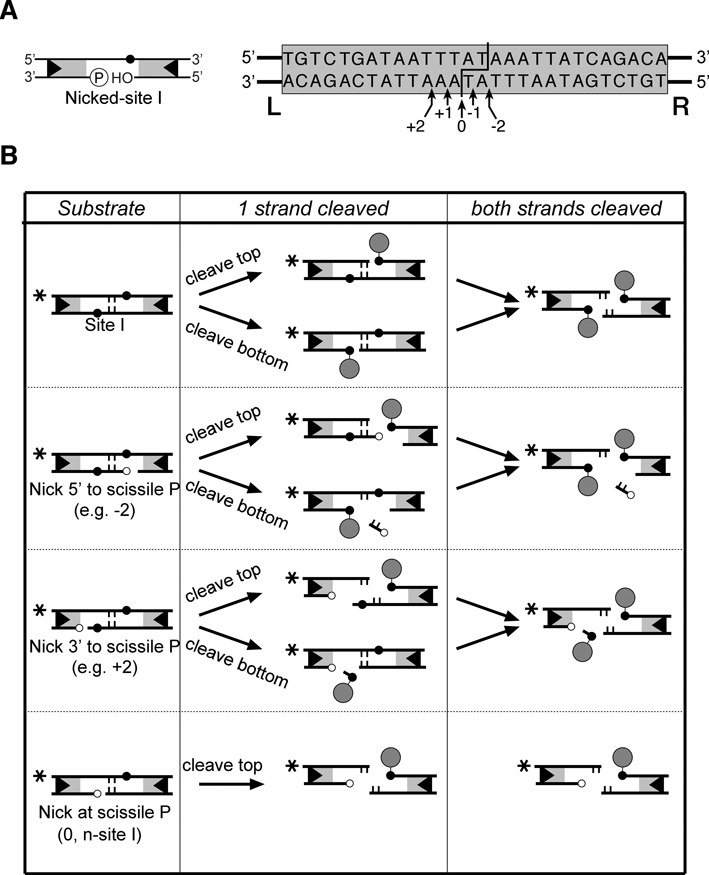
Nicked-site I substrates. (**A**) Left: a cartoon illustrating the generic structure of a nicked-site I. The symbols are as in Figure 1. Right: design of nicked-site I substrates. The site I sequence is shown boxed. The points at which resolvase breaks and rejoins the DNA strands are indicated by the staggered line. The positions of nicks in the bottom strand are indicated by arrows. (**B**) Products of resolvase-mediated cleavage. The substrates used here can be classified into four types as shown in the left-hand column. The products of cleavage of one strand are shown in the centre column, and products of double strand cleavage are in the right-hand column. (The n-site I products are shown again in the bottom panel of the right-hand column, for completeness.) Symbols are as in Figure 1; additionally, the central 2 bp of the sites is shown as short vertical lines, and attached resolvase subunits are shown as filled circles. 5′ phosphate groups at the positions of the nicks are indicated by small unfilled circles. The asterisks indicate the position of the ^32^P radioactive label at the left end of the site as in experiments shown in Figures 3–7.

Binding/synapsis assays were based on previously described methods (7). Briefly, substrates containing site I (the Tn*3* crossover site) were assembled by annealing equimolar amounts of 50 nt (or 70 nt) top and bottom strand oligonucleotides, one of which was first ^32^P-labelled at the 5′ end. The nicked-site substrates used in single-strand cleavage assays were assembled by annealing a 50 nt or a 70 nt site I top strand oligonucleotide with equimolar amounts of two shorter bottom strand oligonucleotides, such that a single-strand break (nick) is present near or at the scissile position on the bottom strand, and the assembled double-stranded substrate has blunt ends. The appropriate oligonucleotide was ^32^P-labelled at its 5′ end with T4 polynucleotide kinase. The top strand of the 70 bp site is 5′-*ggcaagcttg*cgtgactcaacTGTCTGATAATTT**AT**AAATTATCAGACAtagtgggatgg*tctgcagcgg*-3′; the central AT dinucleotide of site I is in bold, and the nucleotides flanking site I are in lower case. The 50 bp site is 10 bp shorter at each end (lacking the nucleotides in italics). The positions of bottom strand nicks are described in the main text. The 5′ end at the nick is phosphorylated unless stated otherwise (see Figure 2A).

An excess of resolvase over DNA sites is required to achieve maximal binding, synapsis, cleavage and recombination in our assays, as has been observed previously for both WT resolvase (8) and activated mutants (7). For binding/synapsis assays, the buffer prior to resolvase addition contained 20 mM Tris-HCl (pH 7.5), 10 μg/ml poly(dI/dC), 4% w/v Ficoll and 50 nM site I DNA. Resolvase, diluted in a buffer containing 20 mM Tris-HCl (pH 7.5), 1 mM DTT, 0.1 mM ethylenediaminetetraacetic acid (EDTA), 1 M NaCl and 50% v/v glycerol, was added (1/10 of the final volume) to give a final concentration of 800 nM resolvase, unless otherwise stated. Samples were incubated at 37°C for 15 min and then loaded onto 7.5% polyacrylamide gels (30:0.8, acrylamide:bisacrylamide). The buffer in the gel and tanks was TBE (100-mM Tris base, 100 mM boric acid, 1 mM EDTA; pH 8.3). Gels were pre-run for 20 min at 150 V, 22°C. After loading the samples, the gels were run for a further 2 h under the same conditions. Gels were dried, and bands were visualized and quantitated by phosphor-imaging.

For DNA cleavage and recombination assays, resolvase (diluted as above; 2.2 μl) was added to 20 μl aliquots containing 25 nM site I (or nicked site I) DNA, 50 mM Tris-HCl (pH 8.2), 10-μg/ml poly(dI/dC) and 8% w/v Ficoll, giving a final resolvase concentration of 800 nM. Reactions were incubated at 37°C and were terminated by the addition of 0.25 volume of Stop buffer (20% w/v Ficoll, 100 mM Tris base, 100 mM boric acid, 1 mM EDTA (pH 8.3), 0.5% w/v sodium dodecyl sulphate (SDS), 1-mg/ml protease K and 0.5 mg/ml bromophenol blue). For analysis of covalent resolvase–DNA adducts, protease K was omitted from the Stop buffer. The samples were analysed on 7.5% polyacrylamide gels (including 0.1% SDS for analysis of resolvase–DNA adducts), as described above. Quantitation of bands on gels was by phosphor-imaging. Curves on the plots shown in Figures 4, 6 and 7 were produced by fitting data points to a simple hyperbolic curve using GraphPad Prism 4. Initial rates of n-site I cleavage (percentage substrate converted per second) were determined from the linear part of the curve. Data points in Figures 4 and 7 are means from three independent replicates of the assay; standard deviation is shown by the error bars.

**Figure 3.**
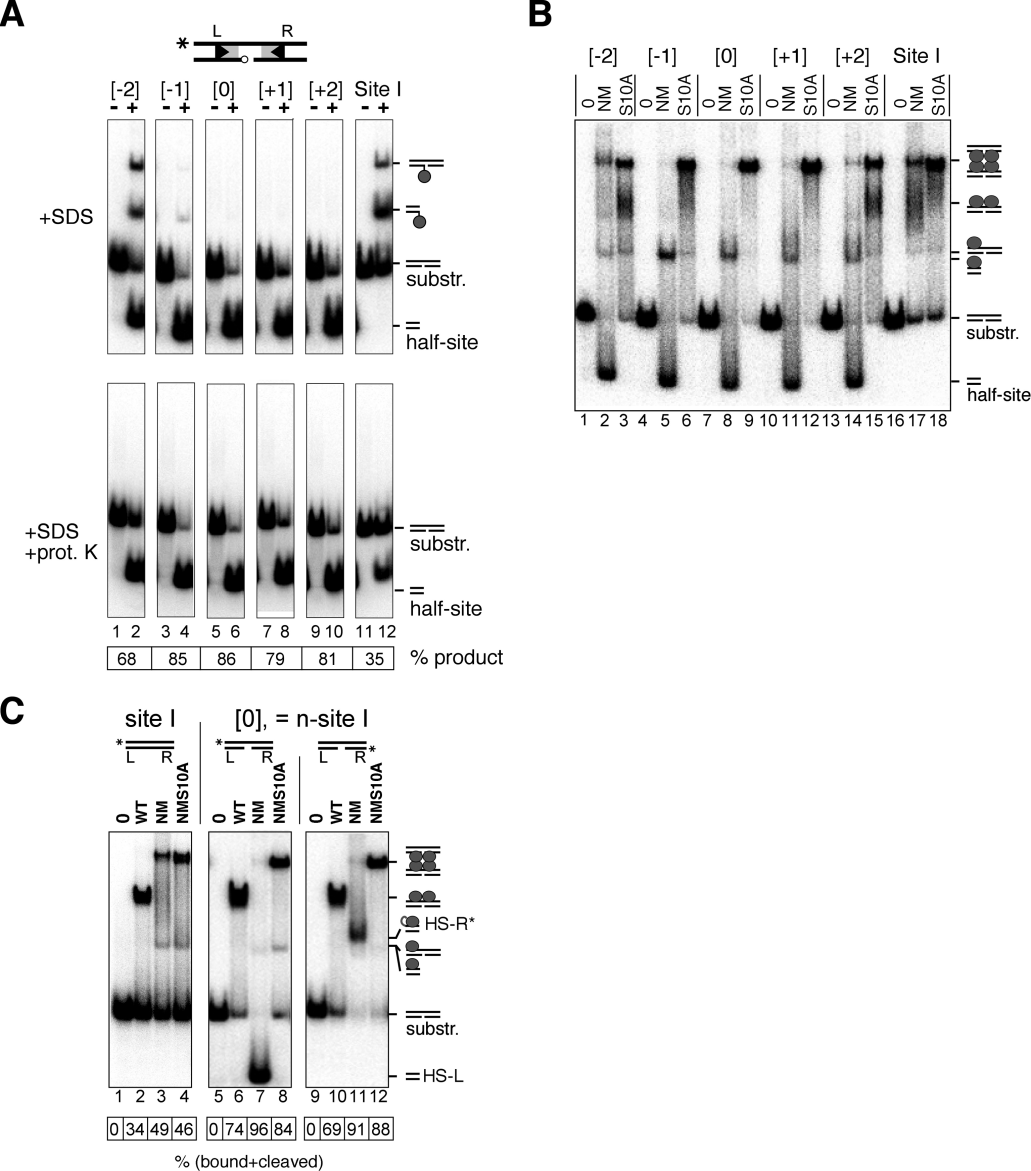
Binding, synapsis and cleavage of nicked-site I substrates by Tn*3* resolvase. (**A**) Substrates (50 bp; 50 nM) were 5′-^32^P-labelled at the left end on the top (intact) strand. They were then treated with the activated Tn*3* resolvase variant NM (400 nM) at 37°C for 30 min. Reactions were stopped by treatment with 0.1% SDS, and products were separated on a 7.5% polyacrylamide gel. The samples in the lower panels were additionally treated with 1 mg/ml protease K prior to electrophoresis. These data were quantitated to give % conversion to half-site product (shown in the box below the gel). The positions of the nicks in the sites designated −2, −1, 0, +1 and +2 are shown in Figure 2A. Assignment of product bands is indicated by the small cartoons on the right of the gel. The reactions leading to these products are illustrated in more detail in Figure 2B. (**B**) Binding, synapsis and cleavage of nicked-site substrates. Substrates (50 bp; 50 nM), labelled as in part (A), were treated with NM resolvase or its catalytically defective mutant NM S10A (both 800 nM), and complexes were separated on a non-denaturing polyacrylamide gel. Complexes with resolvase and reaction products are indicated by the small cartoons to the right of the gel. (**C**) Following the fate of the left and right ends of n-site I. The site I or n-site I substrate (50 bp; 50 nM) was ^32^P-labelled at the left or the right end as indicated by the asterisk in the cartoons above the relevant lanes. The substrates were then treated with resolvase and separated as in part (B). The data were quantitated to give % substrate bound and/or reacted (box below the gel).

**Figure 4.**
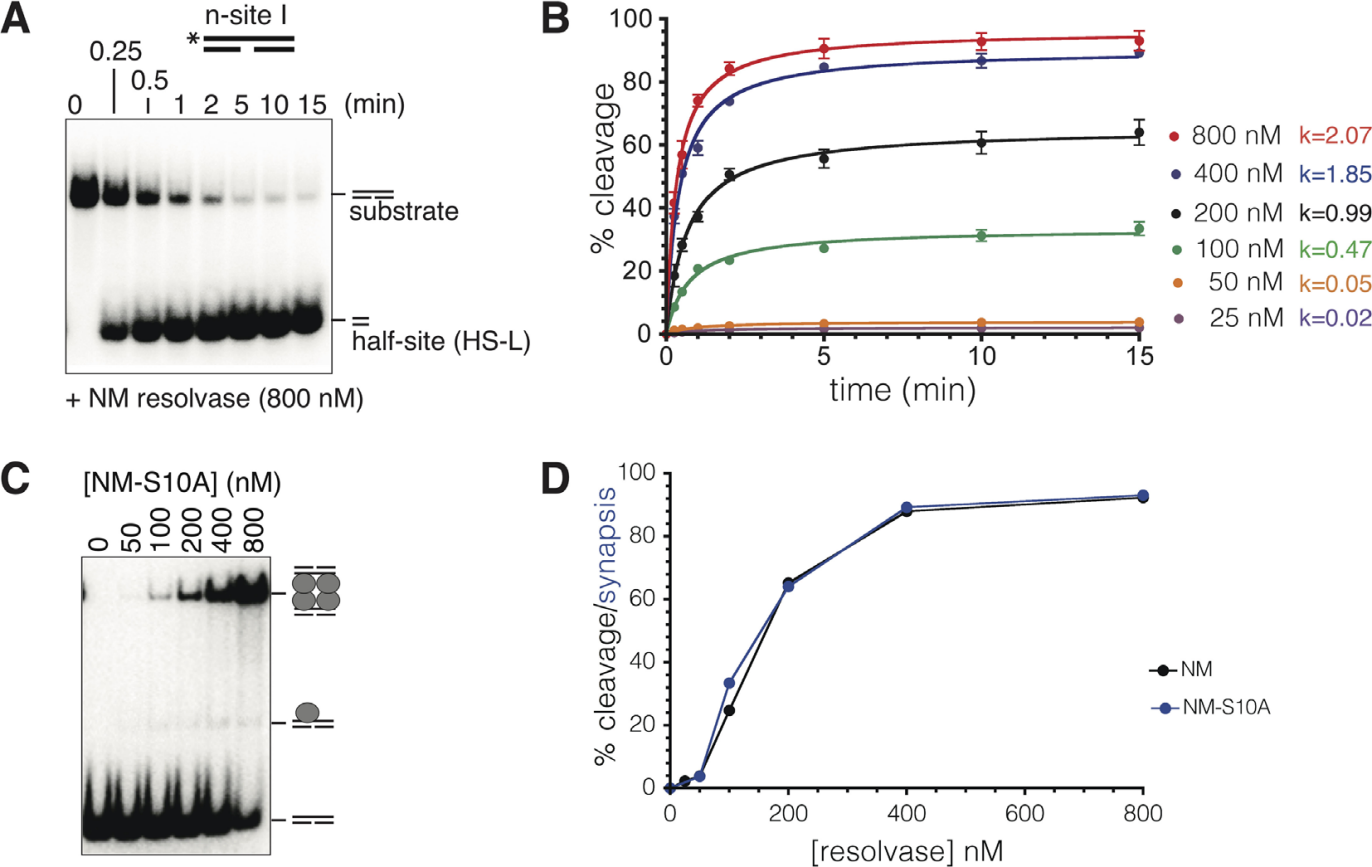
Kinetics of resolvase-mediated n-site I cleavage. (**A**) n-site I (50 bp; 50 nM), 5′-^32^P end-labelled on the intact (top) strand, was treated with NM resolvase (400 nM) at 37°C, aliquots were stopped at the times shown by treatment with 0.1% SDS and the products were separated on a 7.5% polyacrylamide gel containing 0.1% SDS. (**B**) Progress of n-site I cleavage at different NM resolvase concentrations (quantitated from phosphor-images of gels such as that shown in part (A)). The data points are means from three experiments run under identical conditions; error bars indicate standard deviation. Initial rates for each reaction (calculated as % substrate cleavage s^−1^, from the early, linear parts of the curves) are given to the right of the graph. (**C**) Synaptic complex formation. n-site I (50 bp) was treated with different concentrations of NM S10A, a catalytically inactive NM resolvase mutant, and synaptic complex was detected by non-denaturing polyacrylamide gel electrophoresis. (**D**) Concentration dependence of n-site I cleavage after 15 min (black) is compared to the concentration dependence of synapse formation by NM S10A (blue). The cleavage data are the same as those in part (B).

**Figure 5.**
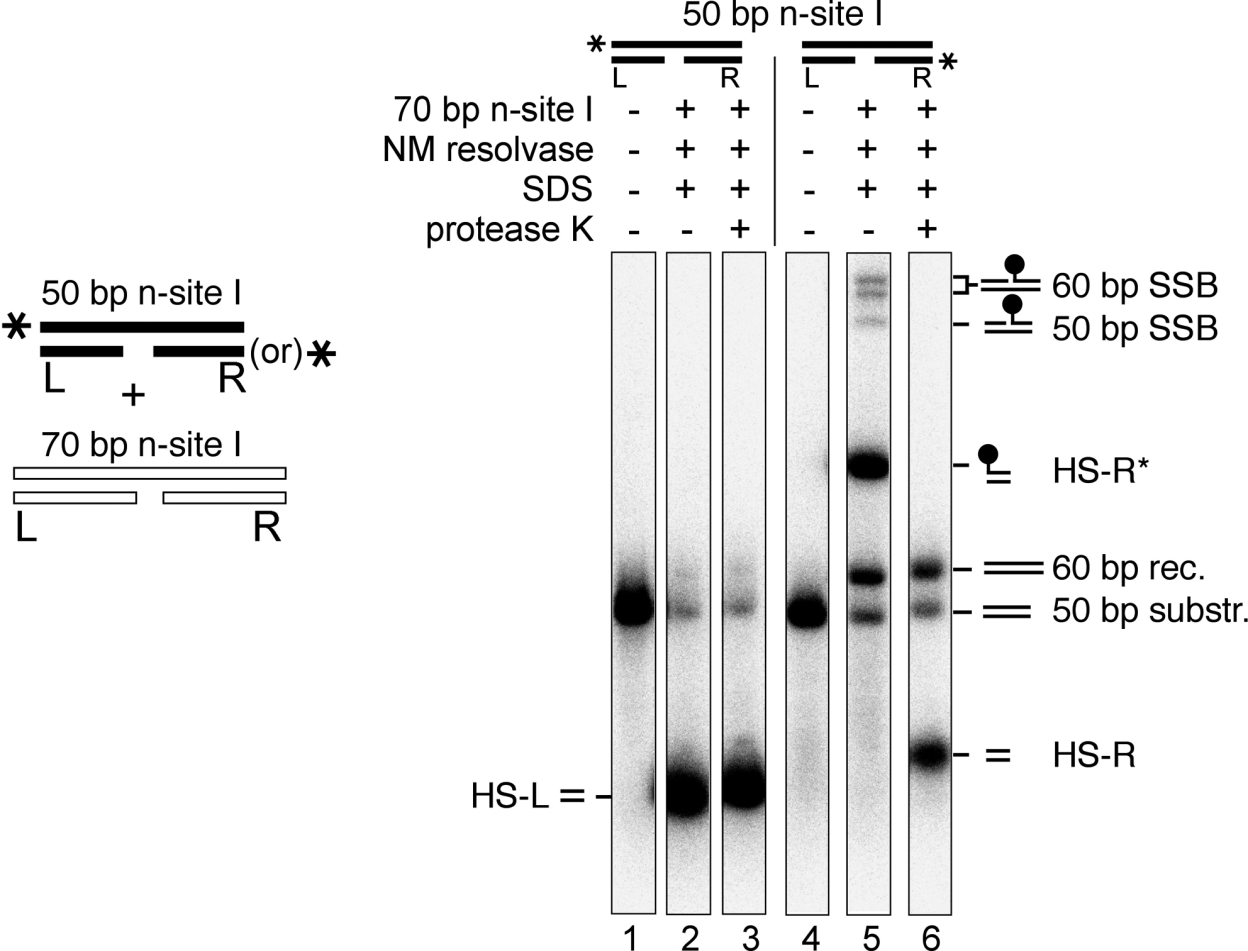
Recombination products of n-site I. The panel on the left summarizes the experimental design; a labelled 50 bp n-site I is reacted together with excess unlabelled 70 bp n-site I. 50 bp n-site I (20 nM), ^32^P-labelled at either the left or the right end as indicated by the cartoons above the lanes, was mixed with an excess (80 nM) of unlabelled 70 bp n-site I and treated with NM resolvase at 37°C for 1 h. Reactions were stopped by treatment with 0.1% SDS (and 1 mg/ml protease K in lanes 3 and 6), and products were separated on an SDS-polyacrylamide gel. Product assignments are indicated by the small cartoons on the right of the gel. SSB, single-strand break product; rec., recombinant. Half-site products HS-L, HS-R and HS-R* are described in the text. The SSB products may be formed by incomplete ligation or strand cleavage of a 60 bp recombinant site, or of a 50 bp ‘recombinant’ site comprising two 25 bp labelled HS-R* half-sites.

**Figure 6.**
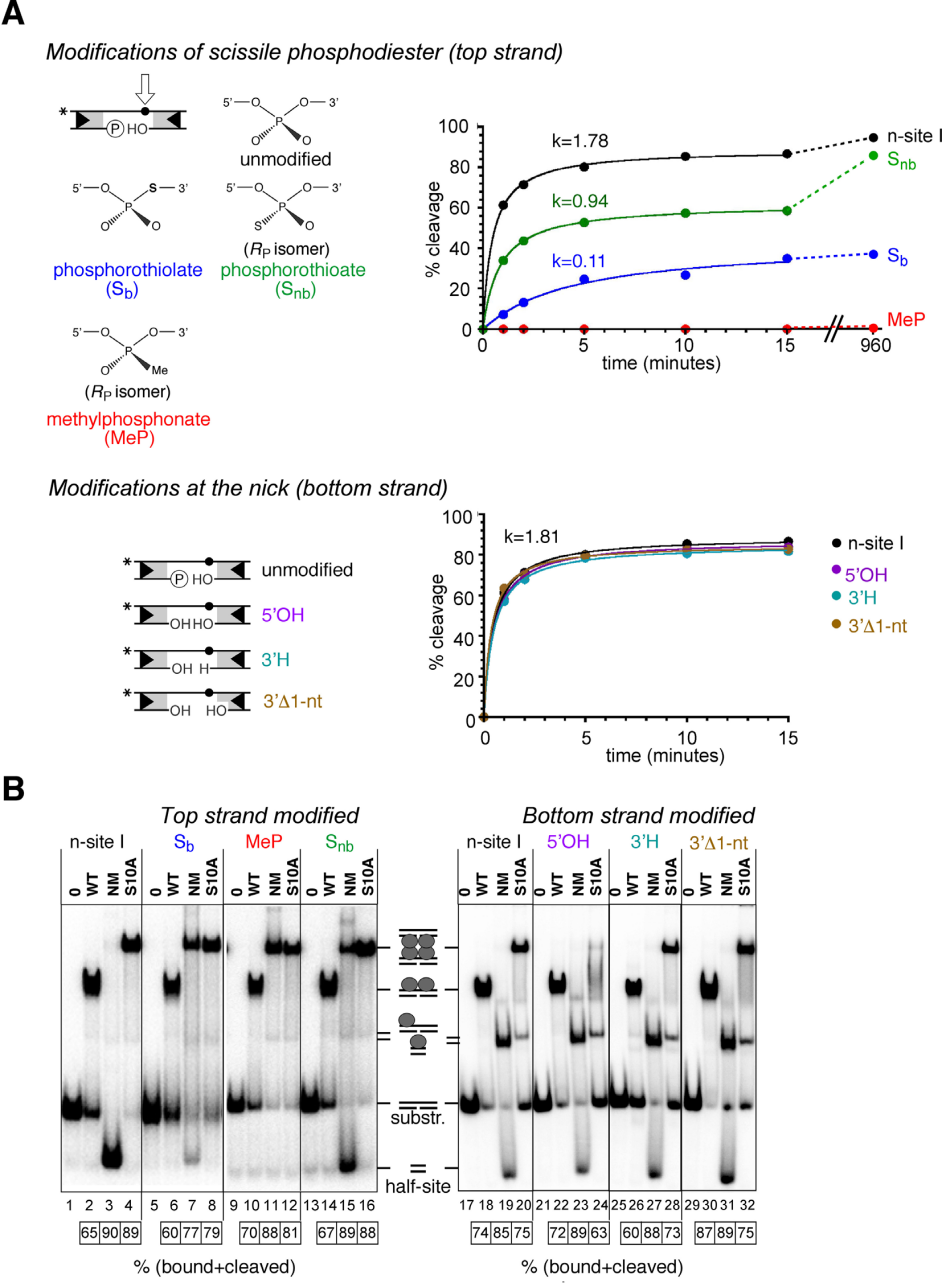
Effects of chemical modifications of n-site I on binding, synapsis and cleavage. (**A**) On the left, structures of the chemical modifications used here are illustrated (further details are given in the Results section). Only the *R*_P_ stereoisomers of the methylphosphonate and phosphorothioate are shown; note that our substrates are ∼1:1 mixtures of *R*_P_ and *S*_P_ stereoisomers. On the right, time courses of NM resolvase-mediated cleavage of the modified n-site I (50 bp) are shown. The NM resolvase concentration was 800 nM, and other reaction conditions were as in Figure 4. Initial rates were calculated as % substrate cleavage s^−1^. (**B**) Effects of n-site I modifications on binding and synapsis. Substrate (50 bp, ^32^P-labelled at the left end) was treated with resolvase variants as indicated above the lanes, and DNA–resolvase complexes were separated by non-denaturing polyacrylamide gel electrophoresis (conditions as in Figure 3). The extents of binding and/or cleavage of the substrates (%) are indicated in the boxes below the gels.

**Figure 7.**
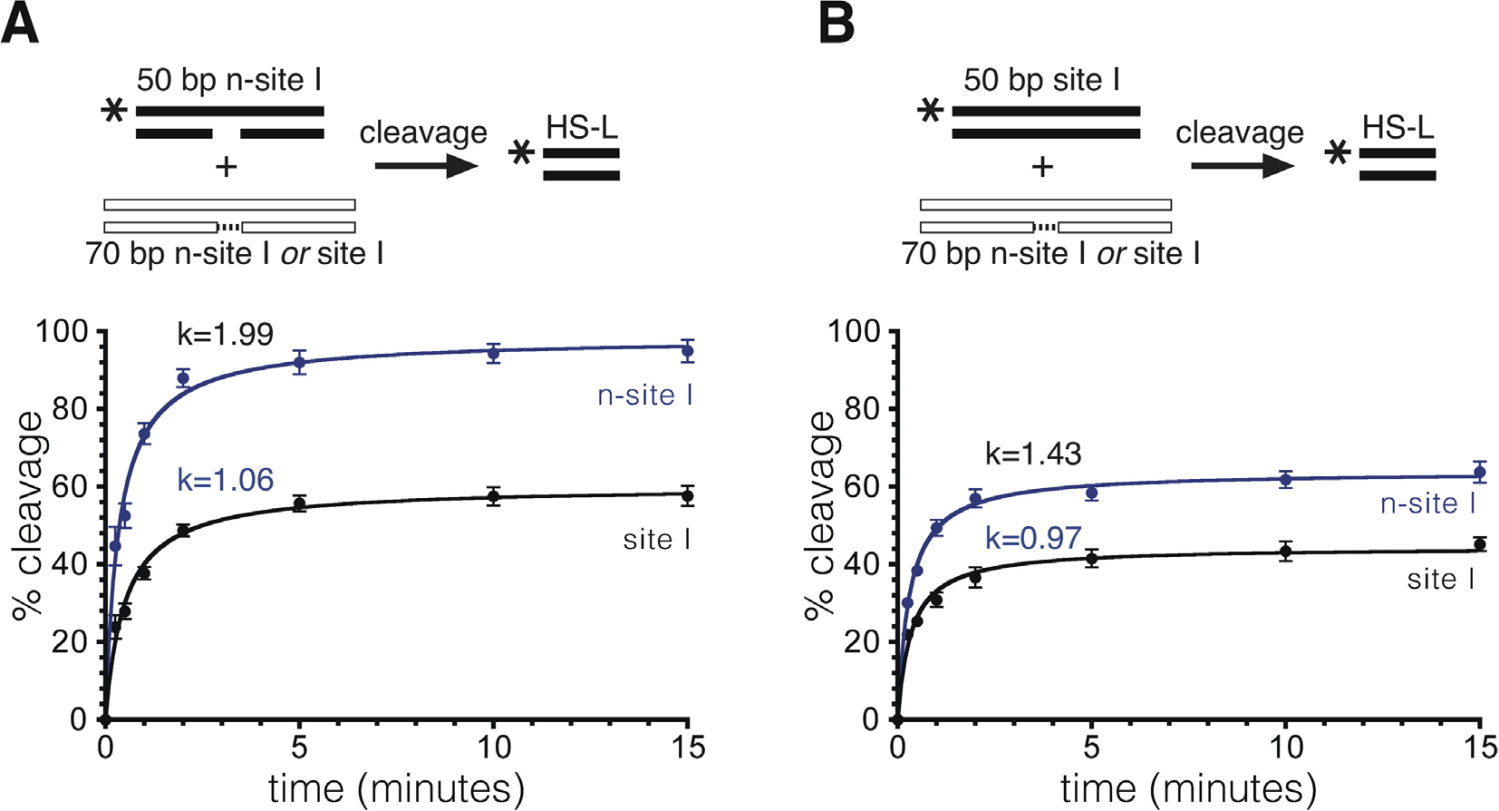
Effect of the partner site on resolvase-mediated cleavage. (**A**) 50 bp n-site I, 5′-^32^P-labelled at the left end (20 nM), was treated with NM resolvase (800 nM) in the presence of excess (80 nM) unlabelled site I (black points and line) or n-site I (blue points and line). Aliquots were stopped at various time points and cleavage was quantified following gel electrophoresis. (**B**) 50 bp site I, 5′-^32^P-labelled at the left end, was mixed with excess unlabelled site I or n-site I and treated with NM resolvase. Cleavage was quantified as in part (A), except that the points represent the sum of cleavage and recombinant products. Data in parts (A) and (B) were from triplicate experiments, as described in the legend to Figure 4B.

The sample buffer contained additional DTT (final concentration 0.5 mM) for all assays with phosphorothiolate-containing substrates.

## RESULTS

### Binding, synapsis and cleavage of nicked-site substrates

The strategy of introducing a single-strand break (‘nick’) in recombination substrates, close to the locations of recombinase-mediated strand cleavage and rejoining, has proved to be very effective for dissection of the mechanistic steps (9–13). Recombination by WT Tn*3* resolvase normally requires a supercoiled substrate and long recombination sites (*res*) containing binding sites for additional resolvase subunits (4), but so-called ‘activated’ resolvase variants have been shown to cleave and recombine ∼30-bp linear crossover sites (‘site I’ of *res*) that each bind a single resolvase dimer (7,14,15). We made nicked-site I substrates for activated resolvase (Figure 2A) by annealing a full-length top strand oligonucleotide with two shorter strands, such that the ‘bottom strand’ has a nick at or close to the position of the scissile phosphodiester (i.e. the phosphodiester attacked by the resolvase active site serine nucleophile). The 5′ end at the nick is phosphorylated and the 3′ end has a hydroxyl group (Figure 2A), so the structure at the nick is identical to that of a single-strand break created by a nuclease such as DNase I or a restriction enzyme. Resolvase-mediated cleavage of the top strand of a nicked site I will sever the only covalent linkage between the left end and the right end of the site, creating two ‘half-sites’, HS-L (left half-site) and HS-R (right half-site), which might dissociate from each other (Figure 2B; see below). Resolvase might also cleave the bottom strand if the nick is close to but not at the scissile phosphodiester (Figure 2B).

The 50-bp substrates, 5′-^32^P-labelled at the left end of the continuous top strand, were incubated with NM resolvase, an activated variant of Tn*3* resolvase (7,15). The reaction products were analysed by polyacrylamide gel electrophoresis after denaturation of the resolvase with SDS. NM resolvase promoted very efficient cleavage of all the nicked-site I substrates to give a ^32^P-labelled HS-L product (Figure 3A, lanes 2, 4, 6, 8 and 10). A control reaction with intact site I gave much less HS-L cleavage product (lane 12). Some substrates gave additional, minor labelled product bands with lower gel mobilities. These bands are predicted to have a covalently attached resolvase subunit as a result of bottom strand cleavage (Figure 2B) and as expected, they disappeared when the resolvase was degraded by protease K (Figure 3A, lower panel).

Activated resolvase variants (such as NM resolvase) form synaptic complexes comprising two copies of site I bridged by a recombinase tetramer, which can be observed by non-denaturing gel electrophoresis (7,16) (Figure 3B, lane 17). However, only traces of intact synaptic complex were observed when the ^32^P-labelled nicked-site I substrates were treated with NM resolvase (Figure 3B, lanes 2, 5, 8, 11 and 14). The predominant labelled product species was cleaved half-site (either protein-free or bound non-covalently by a single resolvase subunit). Synaptic complexes were observed again when NM resolvase was substituted by the catalytically defective NM S10A mutant (Figure 3B, lanes 3, 6, 9, 12 and 15).

### n-site I

We decided to focus our further analysis on the site which has the bottom strand nick exactly at the scissile position ([0]; Figure 2A and B). No bottom strand cleavage reaction of this substrate can take place, because the scissile phosphodiester is already broken. The only viable reaction is top strand cleavage, and this occurs very efficiently (Figure 3A). We call this site hereafter n-site I. We previously used n-site I to assay activity of resolvase mutants (12).

The properties of n-site I were investigated in more detail by ^32^P-labelling it at either the left end or the right end (Figure 3C). We first analysed interactions of WT Tn*3* resolvase with the substrates. WT resolvase binds to site I *in vitro* as a dimer, but does not mediate synapsis of two copies of site I, nor does it catalyse any strand cleavage (7). Similarly, WT resolvase forms dimer complexes with n-site I, but does not make synaptic complexes and has no catalytic activity (Figure 3C, lanes 6 and 10, and data not shown). Interestingly, the affinity of WT resolvase for n-site I is higher than its affinity for intact site I (Figure 3C, compare lane 2 with lanes 6 and 10). NM resolvase gave product bands which depended on the ^32^P label position in n-site I. When the label was at the left end, nearly all of the DNA (96%) was in a high-mobility product band, assigned as free HS-L (Figure 3C, lane 7; see also Figure 3B). When the label was at the right end, the predominant product (91% of total label) was a lower-mobility band assigned as the right half-site (HS-R) with a covalently attached resolvase subunit (designated HS-R*; Figure 3C, lane 11). Only traces of synaptic complex (<5%) were detected with either left end- or right end-labelled n-site I substrates and NM resolvase, whereas the catalytically inactive NM S10A resolvase formed the expected synaptic complex bands in good yield (>80%; Figure 3C, lanes 8 and 12). These results confirm that the n-site I synapse disintegrates or becomes very unstable upon strand cleavage. We hypothesize that this is because HS-L lacks a covalent attachment to resolvase and as a consequence dissociates readily, thereby destabilizing the remaining complex.

### Rate and resolvase concentration dependence of n-site I cleavage

n-site I (25 nM), 5′-^32^P-labelled at the left end (top strand), was treated with a saturating concentration of NM resolvase (800 nM), and samples were taken over a time course. The substrate was rapidly (*t*_1/2_ < 1 min) and efficiently (93% after 15 min) converted to cleavage product (Figure 4A) (12). At lower resolvase concentrations, the reaction was initially fast but the extent of conversion to cleavage product was lower, reaching a plateau (Figure 4B). This incomplete cleavage might be because only a fraction of the total substrate was synapsed and thus available for reaction. The initial rates of the reactions (calculated with respect to total substrate) correlated with the height of the plateau, supporting this hypothesis (Figure 4B). As a further test of this idea, we used the catalytically inactive NM S10A resolvase, which forms a stable synaptic complex (see above). The dependence of synaptic complex formation on NM S10A concentration (Figure 4C and D) was in good agreement with the dependence of cleavage on NM resolvase concentration (Figure 4B and D), supporting the hypothesis that n-site I cleavage takes place only after synapsis of two sites.

### Recombination products from n-site I

As noted above, the two half-sites formed by resolvase-mediated cleavage of n-site I are chemically distinct. The right half-site (HS-R*) is identical to the product of double-strand cleavage of an unmodified site I; it has a resolvase subunit attached by a phosphodiester linkage to the 5′ end of its top strand, and a 3′-OH on its bottom strand (Figure 2B). HS-R* might therefore form a recombinant by ligation with another half-site. The left half-site HS-L has no covalently attached resolvase, and its bottom strand (which bears a 5′ phosphate where a resolvase subunit would normally be attached) cannot be ligated; but in principle HS-L could still react further. Its top strand 3′-OH could attack the resolvase-linked phosphodiester of its partner HS-R* half-site, reversing the cleavage reaction, or it could attack a different resolvase-linked half-site, forming a recombinant product. To detect recombination products, the 50 bp n-site I substrate was ^32^P-labelled at either the left end or the right end and treated with NM resolvase in the presence of excess unlabelled 70 bp n-site I (Figure 5). Reciprocal recombination between the labelled and unlabelled sites would give 60 bp products. A substantial amount of 60 bp product was detected when n-site I was labelled at the right end, but only a trace amount (<1%) was detected when the site was labelled at the left end (Figure 5, lane 3). Therefore, the HS-L cleavage product does not normally react further. Similar results (with slightly lower levels of recombinants) were obtained when the unlabelled partner site was a 70 bp site I instead of n-site I (data not shown).

### Effects of DNA chemical modifications on cleavage

Our analysis above suggests that n-site I is effectively a ‘suicide substrate’; following the only permissible resolvase-mediated reaction (cleavage of the top strand), HS-L dissociates from the reaction complex, rendering cleavage irreversible. Furthermore, top strand cleavage is fast and efficient. Therefore, n-site I is a very suitable substrate to use for investigation of the effects of chemical modifications on serine recombinase-mediated catalysis. Such experiments can reveal details of essential active site–substrate interactions and coordination of steps in phosphoryl transfer reactions (17).

#### Modifications at the scissile phosphodiester

In a **phosphorothiolate**, one of the ‘bridging’ oxygen atoms that link the phosphorus to the deoxyribose carbon atoms is substituted by a sulphur atom (S_b_; Figure 6A). Phosphorothiolates have been used as suicide substrates in phosphoryl transfer reactions. If the sulphur atom substitutes for the normal oxygen leaving group, it is displaced as a thiol or thiolate, and the reverse reaction is unfavourable (13,18–20). We therefore prepared an n-site I substrate with 3′-S_b_ modification of the scissile phosphodiester. However, to our surprise, it was a rather poor substrate; the initial rate of NM resolvase-mediated cleavage was ∼16-fold slower than that of unmodified n-site I (Figure 6A), and the substrate was not cleaved to completion even after 16 h, suggesting that a fraction was trapped in an unreactive state. In contrast, binding and synapsis were not inhibited by the modification (Figure 6B). A substantial amount of synapse was observed with NM resolvase and S_b_-modified n-site I (in contrast to unmodified n-site I; compare lanes 3 and 7), consistent with failure of NM resolvase to promote cleavage and thus destabilize the synaptic complex.

In a **phosphorothioate** modification (S_nb_), a sulphur atom substitutes for one of the ‘non-bridging’ oxygen atoms attached to the scissile phosphorus. The *R*_P_ and *S*_P_ stereoisomers (Figure 6A) are expected to be present in approximately equal amounts in our S_nb_-modified n-site I substrate. S_nb_ modification of the top strand scissile phosphodiester did not affect resolvase binding and synapsis (Figure 7B), but strand cleavage was substantially affected (Figure 7A). The initial cleavage rate was about half that of unmodified n-site I, but product formation reached a plateau at ∼50% of the substrate after a few minutes. Cleavage then continued at a slower rate and was 85% complete after 16 h. Therefore, both S_nb_ stereoisomers are reactive; the data are consistent with the idea that one stereoisomer is cleaved rapidly (about as fast as the unmodified phosphodiester), whereas the other is cleaved much more slowly. The S_nb_-containing cleaved half-site HS-R* was ligatable with other half-sites to form recombinant products (data not shown).

In a **methylphosphonate** modification of a DNA phosphodiester (MeP), one of the non-bridging oxygens on the phosphorus atom is substituted by a methyl group; as a result, a methylphosphonate has no negative charge. Like a phosphorothioate, a methylphosphonate has *R*_P_ and *S*_P_ stereoisomers (Figure 6A), and our MeP-modified n-site I substrate is expected to contain approximately equal amounts of each. Resolvase binding and synapsis were not significantly affected by MeP modification of the scissile phosphodiester (Figure 6B), but there was no observable cleavage by NM resolvase, even after incubation for 16 h (Figure 6A). Both methylphosphonate stereoisomers are therefore inert.

#### Modifications at the bottom strand nick

In all the nicked-site substrates reported above, the 5′ DNA end at the nick is phosphorylated, and the 3′ end has a hydroxyl group. We investigated the effects of simple modifications of the DNA at the nick in n-site I. The nick in the DNA and the nature of the 5′ DNA end at the nick affect the charge at this position. The natural phosphodiester has one negative charge, whereas the 5′ phosphate in n-site I has two negative charges; an n-site I substrate with no 5′-phosphate (5′-hydroxyl) has zero charge. Substitution of the 5′ phosphate with a hydroxyl group (5′ OH) had no observable effect on binding by WT resolvase, but the amount of synaptic complex formed with the catalytically defective NM S10A resolvase was reduced, suggesting a minor perturbation of resolvase–DNA interactions (Figure 6B). The rate of NM resolvase-mediated cleavage was also very slightly reduced (Figure 6A). We also modified the 3′ DNA end at the nick position by replacing the 3′-hydroxyl group with a hydrogen atom (3′H). Binding of WT resolvase, synapsis by NM S10A resolvase and strand cleavage by NM resolvase were only slightly reduced by 3′H modification (Figure 6). Even a more substantial modification, complete removal of the 3′-terminal nucleotide at the nick (3′Δ1), had very small effects on binding, synapsis and cleavage (Figure 6).

### Communications between sites in the synapse

The efficient irreversible cleavage of n-site I provides a useful tool to analyse the coordination of strand cleavage events within the synapse. We can ask the question: Is cleavage at one site affected by cleavage of the partner site? An n-site I substrate (^32^P-labelled at the left end; 20 nM) was mixed with an excess (80 nM) of an unlabelled site, so that most labelled n-site I would be synapsed with an unlabelled partner site. The unlabelled site was either n-site I, which is cleaved rapidly, or site I, which is cleaved more slowly (7). Cleavage of the labelled n-site I was higher when the unlabelled partner site was also n-site I (Figure 7A), suggesting that cleavage of one site stimulates cleavage of the other. We also carried out analogous experiments in which labelled site I was synapsed with an unlabelled site I or n-site I partner. Here, formation of a labelled half-site product requires cleavage of both top and bottom strands of intact site I. Site I cleavage was more efficient when the partner site was n-site I than when it was site I (Figure 7B). We conclude that double-strand cleavage of one site within a synapse stimulates cleavage of the partner site.

## DISCUSSION

### Resolvase–DNA interactions

A nick in one of the DNA strands at any of several positions close to the scissile phosphodiester in site I has a dramatic effect on the products of resolvase-mediated reactions (Figure 3). All the substrates that we tested gave large amounts of species consistent with failure to complete recombination. A major product was a half-site that has no covalent attachment to resolvase. We hypothesize that this half-site dissociates readily from the cleaved reaction intermediate and thus becomes unavailable for any further reactions. Consistent with this hypothesis, we observe much reduced levels of synaptic complexes with nicked-site substrates, whereas substitution of NM resolvase by a catalytically inactive mutant (NM S10A) restores efficient synaptic complex formation (Figure 3B). The covalent linkage of DNA half-sites to resolvase subunits thus has an essential role as a tether to prevent escape of the cleaved DNA ends from the reaction intermediate. During strand exchange by subunit rotation, each half-site must move together with its attached resolvase subunit (Figure 1). The complex of a single subunit with its half-site without covalent protein–DNA linkage is thus apparently too unstable to endure through the rotation step until re-ligation. Tn*3* resolvase is predominantly monomeric in solution and binds cooperatively to form dimers on site I DNA. The dimer dissociation constant *K*_d_ is ∼40 nM (21), so the *K*_d_ of a monomer might be substantially higher, consistent with a short lifetime of the monomer–DNA complex.

The rate-limiting steps in resolvase-mediated reactions are after synapsis, which has been shown to be very fast (*t*_1/2_ < 1 min at 0°C; (7,8)). Previous analyses support the idea that the strand cleavages at a single site are coordinated; relatively slow (probably reversible) first strand cleavage is followed by faster second strand cleavage, yielding predominantly DNA double-strand break products (20,22–25). n-site I cleavage is faster (*t*_1/2_ < 1 min at 37°C) than any other resolvase-mediated enzyme activity that we have observed; in similar assays, single-strand cleavage of site I is apparently much slower (*t*_1/2_ ∼ 15 min). Cleavage of n-site I might therefore be analogous to second strand cleavage of site I (the nick mimicking prior cleavage of the bottom strand). Resolvase has higher affinity for n-site I than for site I (Figure 3B). We hypothesize that binding of a resolvase dimer to site I might cause tension in the DNA (bending and/or twisting) which is relieved by first strand cleavage (or the n-site I nick), allowing easier assembly of the active site around the scissile phosphodiester of the second strand and thus faster cleavage.

Our results also indicate that double-strand cleavage of one of the sites in a synapse stimulates cleavage of the other site (Figure 7). There is therefore communication between the two sites in a synaptic complex undergoing strand exchange, presumably mediated by conformational changes in the catalytic resolvase tetramer.

### Resolvase interactions with the DNA scissile phosphodiesters

**Figure 8.**
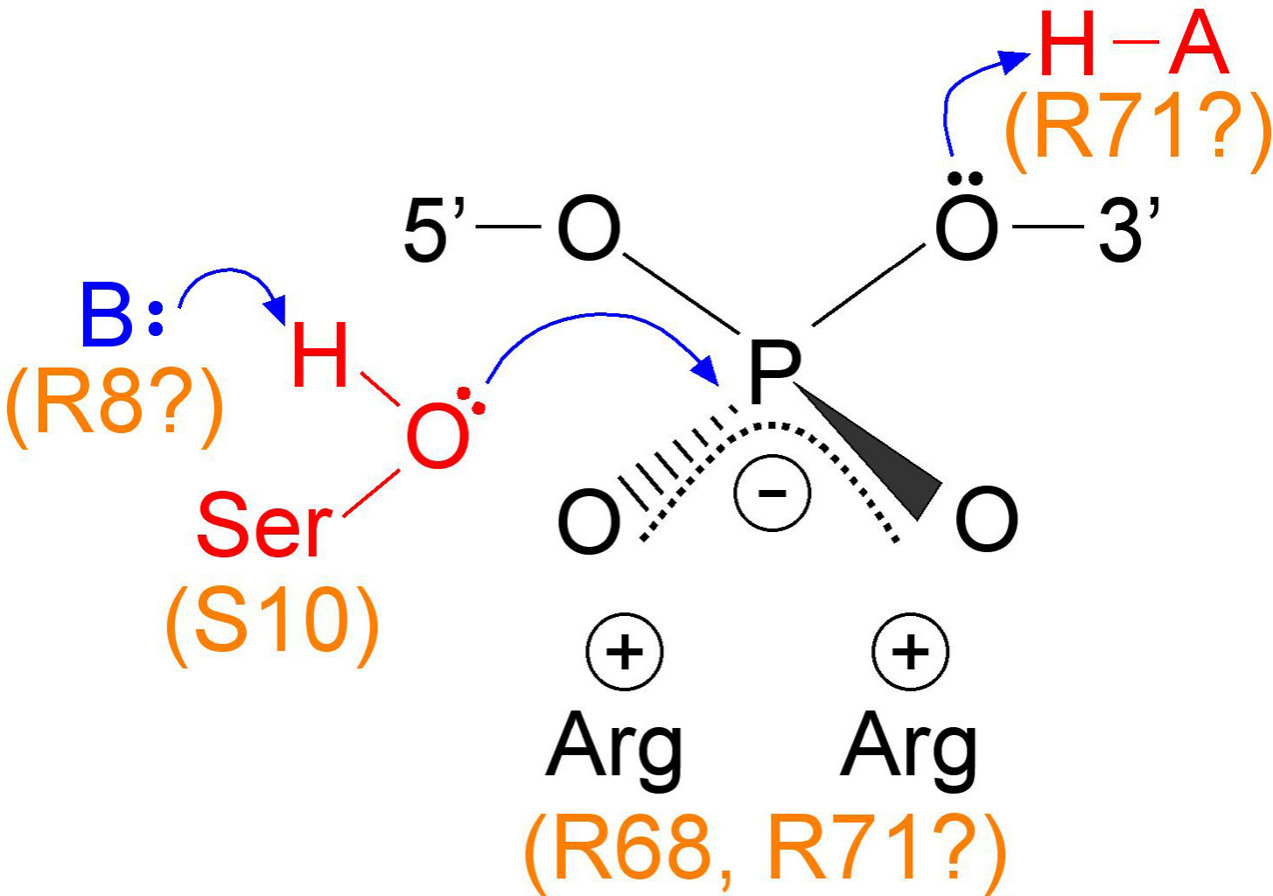
Hypothesized interactions of serine recombinase active site residues with the scissile phosphodiester. A general base (B) deprotonates the nucleophilic hydroxyl group of the active site serine residue, and a general acid protonates the 3′-oxygen atom during strand cleavage. Negative charge on the phosphodiester non-bridging oxygens is balanced by the positively charged sidechains of arginine residues. Candidate Tn*3* resolvase residues for these roles are indicated.

The catalytic mechanism of serine recombinases is expected to have features in common with the mechanisms of tyrosine recombinases and other phosphoryl transfer enzymes (26–28). The active sites of these enzymes typically include a base to remove a proton from the nucleophile(s), and an acid to protonate the leaving group(s). Another universal feature is coordination of substrate non-bridging phosphate oxygen atoms by electropositive groups. These interactions may increase the positive charge on the phosphorus atom, making it a stronger electrophile, and stabilize the double negative charge of the pentacovalent transition state (26). Metal ions, especially Mg^2+^, perform this function in many enzyme active sites, but serine recombinases have no obligatory metal ion requirement; instead, it is proposed that sidechains of conserved arginine residues coordinate the non-bridging oxygens (12,13,20,29–31) (Figure 8). Current crystallographic structures of resolvase–DNA complexes reveal contacts of two arginine residues (corresponding to Tn*3* resolvase R8 and R68) with the non-bridging oxygens of the scissile phosphodiester, but a third arginine, R71, is also proposed to make contacts in the fully formed active site complex (13).

Our experiments indicate that resolvase catalytic activity is easily perturbed by chemical modifications of the scissile phosphodiester. Phosphorothioate (S_nb_) modification substitutes one of the non-bridging oxygen atoms with a larger sulphur atom (Figure 6A), and might thus perturb coordination by active site residues. The kinetics of S_nb_-modified n-site I cleavage (Figure 6A) suggest that one phosphorothioate stereoisomer is a much better substrate than the other, as has been observed in other systems (32,33), but current structural data do not lead to an obvious prediction of which stereoisomer should be preferred by resolvase. A methylphosphonate (MeP) is a more drastic modification of the phosphodiester which replaces one of the non-bridging oxygens with a larger methyl group and has no negative charge. The effects of MeP modification on coordination by active site residues might therefore be both steric and electronic. Tyrosine recombinases and Type IB topoisomerases accept both *R*_P_ and *S*_P_ methylphosphonate stereoisomers as substrates for catalysis, albeit with reduced rates (34,35). In contrast, cleavage of n-site I was completely abolished by MeP modification at the scissile position, so neither stereoisomer is a substrate. This result is consistent with essential contacts by resolvase active site residues to both non-bridging oxygen atoms.

A 3′-S-phosphorothiolate modification (S_b_) of the n-site I scissile phosphodiester substitutes the ‘bridging’ 3′-oxygen with a sulphur atom. This oxygen atom is the leaving group when resolvase promotes strand cleavage by attack of its active site serine hydroxyl group on the scissile phosphodiester. Phosphorothiolate substrates were used previously to probe the mechanism of the Sin serine recombinase (13,20). Resolvase binding and synapsis were apparently unaffected by S_b_ modification, but cleavage was much reduced (Figure 6). S_b_ modification was also reported to inhibit DNA cleavage by the restriction endonuclease EcoRV (36). It was proposed that the bulky sulphur atom interfered sterically with interactions between catalytic metal ions and the phosphodiester non-bridging oxygen atoms. Our results might be explained similarly, if sulphur substitution perturbs interactions with active site arginine residues (see above), either by its increased steric bulk or by weakened hydrogen bonding interactions. Alternatively, the modification might perturb direct contacts of resolvase (or a bound water molecule) with the 3′ leaving group. Current crystallographic data of serine recombinases in complexes with DNA (see, for example, (29,30,37)) do not report any direct contacts with the 3′ oxygens of the scissile phosphodiesters, which might be expected to involve the general acid proposed to protonate the oxygen atom to make it a better leaving group (Figure 8; (13)).

Cleavage of n-site I was most efficient when there was a 5′ phosphate and a 3′-OH at the nick in the bottom strand. However, several modifications of the DNA at the nick position did not have any dramatic effects on resolvase, binding, synapsis or cleavage (Figure 6). We conclude that cleavage of the n-site I top strand does not depend on specific resolvase interactions with the site of prior cleavage of the bottom strand. We hypothesize that n-site I reactions mimic second strand cleavage (see above), so these results suggest that in a standard substrate, cleavage of the second strand does not require continued engagement of the resolvase active site with the already-cleaved strand.

## CONCLUSIONS

Our studies with nicked-site I substrates reveal fast, efficient resolvase-catalysed cleavage of a single DNA strand, which we propose to be masked by rapid reversibility (re-ligation) in unmodified recombination sites. The facile, irreversible dissociation of a half-site cleavage product from the resolvase–DNA synaptic intermediate in our reactions implies that covalent tethering of the cleaved half-sites to resolvase is essential for preservation of the structural integrity of the natural intermediate. Our experiments indicate communications between the two sites in the synaptic intermediate as well as between the two strands of a single site. The effects of substrate chemical modifications imply specific contacts of resolvase active site residues with the scissile phosphodiesters, consistent with current crystallographic and mutational data.
